# Dr. Drake’s Treatise

**Published:** 1850-06

**Authors:** 


### DR. DRAKE'S TREATISE.

The first volume of Dr. Drake's long expected work has at length made its appearance, under the title of "*A Systematic Treatise, Historical, Etiological, and Practical, on the Principal Diseases of the Interior Valley of North America, as they Appear in the Caucasian, African, Indian, and Esquimaux Varieties of its Population,*" and, in advance of a more extended notice which we hope to prepare for our next number, we hasten to give our readers our first impressions of the work. We have for a number of years been familiar with the plan of this treatise, having seen several of its first chapters in manuscript. We have been apprised of the extraordinary labor of its author in collecting the materials of which it is composed. We fully understood, all along, that it was no part of his design to compile a treatise from the published works of others, but to explore for himself the sources of disease in the great interior valley, and to record his own experience and that of his brethren, touching the disorders incident to that region. The plan is an enlarged one and imposed a task of vast magnitude upon its author. In the fulfilment of it, he has found it necessary to visit every quarter of this extended region, from the Gulf of Mexico to the Lakes, and from the brow of the Appalachians as far West as civilization has reached towards the Rocky Mountains. Thus aware of the energy and laborious industry with which its gifted author was devoting himself to his work, we have looked forward to its completion with deep interest and with large expectations.

These expectations Dr. Drake has fully met in the first volume of his elaborate work. Without pretending to assert that it is as perfect as it might have been rendered, we take great pleasure in saying that it is a volume which, in our judgment, does honor, not only to its author, but to our country. Errors will be pointed out in it which future editions will not contain; and in time, upon the foundation which it has laid, other treatises marking the progress of medical science will appear; but the time will hardly come when it will not be quoted by writers on the diseases of America. Himself a pioneer, Dr. Drake is fortunate in having written a book which must remain long a guide to those who shall come after him in this field.

We have not room, at present, to particularize, but will merely add, that Dr. Drake has produced an original work. The contents of this volume are such as can be found in no other volume besides. In it he has given to the profession the results of what he himself has seen and experienced, or what has been observed by others in the region to which his treatise relates; and as a record of original observations it is unrivaled by any work on medicine yet written in our country. There is no need that we should recommend such a work to our readers; they will be in haste to procure it, and to possess their minds of its rich scientific stores. Although but the first of two or three volumes, this volume is complete in itself and would not be less interesting or less valuable if the remainder should never appear. We are glad, however, to know that the indefatigable author is still industriously engaged upon the work, and that most of the materials for its consummation are already collected. Y.

